# Efficacy of physiotherapy treatments in children and adolescents with somatic symptom disorder and other related disorders: systematic review of the literature

**DOI:** 10.1186/s13052-022-01317-3

**Published:** 2022-07-27

**Authors:** Roberta Sartori, Antimo Tessitore, Aurora Della Torca, Egidio Barbi

**Affiliations:** 1 IRCCS Materno Infantile Burlo Garofolo, Trieste, Italy; 2 University of Trieste, Piazzale Europa, 1, 34127 Trieste, Italy

**Keywords:** Functional somatic symptoms, Psychogenic, Medically unexplained physical symptoms, Conversion disorders, Physiotherapy rehabilitation, Paediatric

## Abstract

According to the latest version of the Diagnostic and Statistical Manual of Mental Disorders, somatic symptom and related disorders (SSRDs) are defined as psychopathological manifestations characterized by physical signs not attributable to organic pathology. Their incidence has grown dramatically over the past few decades, and treatment is challenging. Besides other interventions on the child and the family, physiotherapy is considered an integral part of the treatment, although there is no evidence for its efficacy.

The study aimed to review the available proof on the effectiveness of physiotherapy in children and adolescents with SSRDs. A systematic literature search was conducted on MEDLINE/PubMed, CINAHL, Cochrane Library, PsycINFO, and PEDro, including 1999 to 2021. The methodological quality of the publications was assessed by applying the guidelines proposed by the Equator network, according to the different study designs. The scientific bibliography on the subject was minimal and had poor methodological quality. The choice of outcome indicators and the scales to measure them varied from study to study and were not standardized, making comparison and meta-analysis challenging.

**Conclusion:** According to the available evidence, it is impossible to answer the review question regarding the effectiveness of physiotherapy in children and adolescents with SSRDs. It is necessary to improve the methodological quality of the studies. Definition of standard rehabilitation treatments, identification of appropriate result indicators, and adoption of standardized evaluation scales are needed.

## Introduction

The current version of the Diagnostic and Statistical Manual of Mental Disorders (DSM-5) defines SSRDs as a set of psychopathological manifestations characterized by physical symptoms not attributable to organic pathology. The common feature of these disorders is the symptoms prominence neither intentionally produced nor simulated; some medical conditions may be present; however, this can explain neither the intensity nor the pathophysiological mechanisms [1].

These diseases incidence is growing sharply, especially in childhood and adolescence, with a higher prevalence in the female population.

SSRDs include somatic symptom disorder (SSD), conversion (functional neurological symptom disorder), illness anxiety, factitious disorder, psychological factors affecting other medical conditions, additional specified somatic symptoms and related illnesses, unspecified somatic symptoms, and related disorder.

The research examined only the disorders where physiotherapy treatment is reported and indicated as somatic symptoms, conversion disorder, and fictitious disturbances.

Somatic Symptom Disorder is defined based on three criteria and three specifiers described in Table 1. Diagnostic criteria for conversion and fictitious disorders are shown in Tables 2 and 3.

**Table 1:** The diagnostic criteria for Somatic Symptom Disorder noted in DSM 5

A. One or more somatic symptoms that are distressing or result in significant disruption of daily life
B. Excessive thoughts, feelings, or behaviours related to the somatic symptoms or associated health concerns as manifested by at least one of the following: Disproportionate and persistent thoughts about the seriousness of one’s symptoms. Persistently high level of anxiety about health or symptoms. Excessive time and energy devoted to these symptoms or health concerns.
C. Although any somatic symptom may not be present continuously, the state of being symptomatic is persistent (typically more than 6 months).
Specify if: ** With predominant pain** (previously pain disorder): This specifier is for individuals whose somatic symptoms predominantly involve pain. ** Persistent**: a persistent course is characterized by severe symptoms, marked impairment, and long duration (more than 6 months).

**Table 2:** The diagnostic criteria for Conversion Disorder noted in DSM 5

A. One or more symptoms of altered voluntary motor or sensory function.
B. Clinical findings provide evidence of incompatibility between the symptom and recognized neurological or medical conditions.
C. The symptom or deficit is not better explained by another medical or mental disorder.
D. The symptom or deficit causes clinically significant distress or impairment in social, occupational, or other important areas of functioning or warrants medical evaluation.
Specify symptom type: With weakness or paralysis With abnormal movement With swallowing symptoms With speech symptom With attacks or seizures With anaesthesia or sensory loss With special sensory symptom With mixed symptoms
Specify if: Acute episode: Symptoms present for less than 6 months. With psychological stressor: (specify stressor). Without psychological stressors.

**Table 3:** The diagnostic criteria for Factitious Disorder noted in DSM 5

A. Falsification of physical or psychological signs or symptoms, or induction of injury or disease, associated with identified deception.
B. The individual presents himself or herself to others as ill, impaired, or injured.
C. The deceptive behaviour is evident even in the absence of obvious external rewards.
D. The behaviour is not better explained by another mental disorder, such as delusional disorder or another psychotic disorder.
Specify: Single episode Recurrent episodes (two or more events of falsification of illness and/or induction of injury)

Currently, there is a lack of precise data referring to the European situation, but according to a recent American study, these disorders cost the US health system up to 100 billion dollars a year [2, 3].

The role of poor physical activity as a risk factor in developing SSRDs is well known. A study [4] revealed a strong correlation between a low level of physical activity (b = 0.005, bootstrap 95%-IC: 0.01 to 0.09) in a cohort of 1816 adolescents with SSD, a sedentary life (b = 0.10, bootstrap 95%-CI: 00.6 to 0.14) and the onset of the disorder. Furthermore, in a consolidated pragmatic perspective, physicians are aware of the protective role of physical activity in preventing SSD. Besides, regular physical exercise can also facilitate social relationships, avoiding isolation, an additional well-known risk factor [5]. A recent study reported that 10% of patients with mental health problems entered the hospital using walking devices such as wheelchairs or crutches not required by any clinical diagnosis [6]: these patients had a greater need for multidisciplinary support with paediatricians, child psychiatrists, psychologists, nurses and physiotherapists [7].

The physiotherapist is considered an essential role within the multidisciplinary team for these reasons. However, the proposed interventions are very heterogeneous, and the evidence of their efficacy is limited [8].

This systematic review aims to evaluate the available evidence on the effectiveness of physiotherapy treatments in the paediatric population affected by SSRDs.

## Materials and methods

### Search strategies

For this review, we conducted a literature search on the following electronic databases: MEDLINE / PubMed, CINAHL, Cochrane Library, PsycINFO, and PEDro, using the terms listed in Table 4, split according to the population, pathology, and treatment groups.

**Table 4:** Keywords used for database searches

*Population*	*Pathology*	*Treatment*
“pediatric” “paediatric” “child” “children” “adolescent” “adolescence” “minor” “infant”	“functional somatic symptoms” “somatoform disorders” “somatoform” “somatic symptom disorder” “functional weakness” “functional overlay” “psychogenic” “functional disorders” “medically unexplained physical symptoms” “conversion disorders” “functional neurological symptom disorder” “other specified somatic symptom and related disorder”	“physiotherapy” “physioterap*” “exercise” “exercise therapy” “exercise program*” “exercise regime*” “graded exercise therapy” “rehabilitation” “physical therapy” “physical therap*” “non-pharmacological therapies”

### Inclusion and exclusion criteria

The inclusion and exclusion criteria employed to select the articles are described in Table 5.

**Table 5:** Inclusion and exclusion criteria

Inclusion criteria	Exclusion criteria
- Age between 8 and 21 years - Subjects belonging to the category of somatic symptom disorder and other related disorders (SSRD) - Studies published in English and Italian language - Articles in which physiotherapy interventions are cited, and objectives are defined	- Chronic general pain, anxiety disorder, psychological factor disorder affecting other medical conditions - Studies that did not state the results in terms of the effectiveness of the rehabilitation treatments - Articles whose target population was affected by an anxiety disorder and psychological factors that influence other medical conditions

Due to limited publications and poor methodological quality, we considered all research in electronic databases from 1999 to October 2021. Regardless of the study design, we selected all the papers written in Italian and English in full-text form.

Studies with a population of patients aged 8 to 21 years with SSRD disorders (defined according to DSM-5 criteria) requiring physiotherapy treatment, i.e., somatic symptom disorder, conversion disorder, and factitious disorder, were included. Research that considered illness anxiety disorders, disorders with psychological factors affecting other medical conditions, and publications dealing with the definition of “general chronic pain” in children were excluded.

Only the publications in which physiotherapy treatment was cited and described, albeit briefly, in its objectives and methods were examined, while research that did not indicate efficacy results was ruled out.

### Selection of studies

**Fig. 1:**
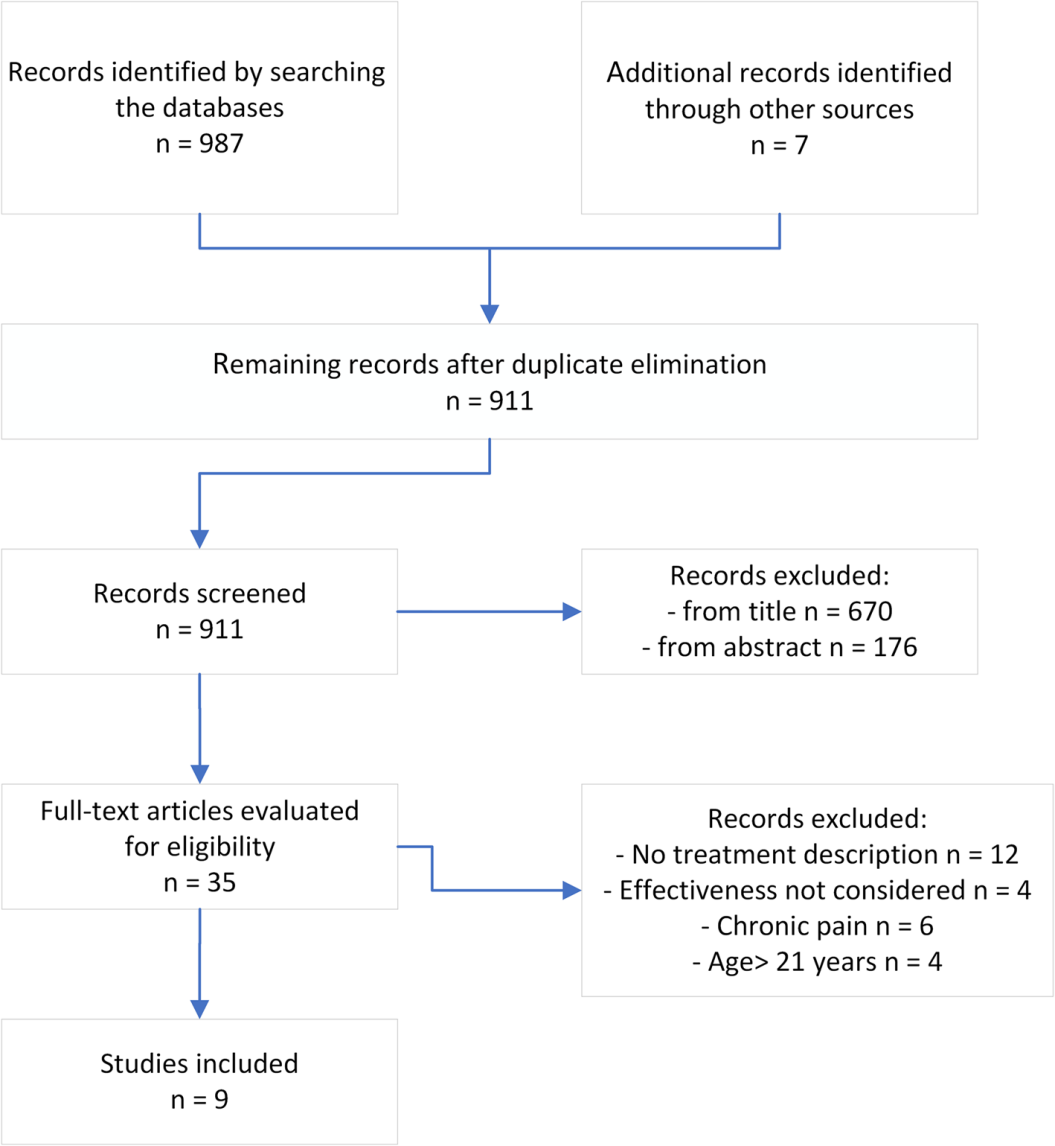
Study selection flow chart

By searching the databases with the keywords mentioned above, 987 studies were selected, with seven additional publications taken from the reference bibliographies of some chosen articles (Fig. 1). Thirty-six full-text articles were evaluated for eligibility, and only 9 met the inclusion criteria. Twelve publications were rejected as they did not describe the objectives and methods pursued by the rehabilitation treatment. We excluded four works that did not consider the effectiveness of the treatments described, and six studies were dropped due to patients with symptomatic manifestations that did not meet the diagnostic criteria of the DSM-5. Finally, four articles were ruled out because they included subjects over 21 in the study population.

The articles were searched between July and September 2021 and involved two independent reviewers, R.S. and A.D.T., who simultaneously selected the papers starting from title and abstract following the inclusion and exclusion criteria. A third reviewer, R.C., resolved any disagreements.

### Characteristics of the studies

The characteristics of the studies examined are described in Table 6. The age of the participants varied from 0 to 21 years. In five studies [9–13], treatments were proposed during hospitalization, while only one was chosen as an outpatient setting [14]. An initial course of treatment was performed in the hospital in the remaining three, followed by outpatient treatment [8, 15, 16]. Treatments and hospitalizations' duration was very variable, [8, 9, 11] ranging from 3 to 78 days of hospitalization, [9] with a duration of treatment from three days to two years [8]. Furthermore, it was not reported, except by an author, [16] whether the treatment started in hospitalization continued after discharge, following specific modalities, intensity, and setting [9–12, 17]. Few papers indicated the intensity of the treatments, regarding the duration of the single session [12] and the frequency of treatments. Follow-up times varied considerably from two months [12, 16] to one year [12], and in three studies, the duration was not indicated [11, 14, 17]. Five of the investigations analyzed were case reports and retrospective case series [10–12, 14, 16], three were observational studies, [9, 15, 17] one was a systematic review [8], Most of the reviewed publications assessed a paediatric population with conversion disorder [8, 11, 12, 14, 17], two included somatic symptom disorder [10, 15], one examined SSRDs [16], and finally, in one article, the diagnosis was nonspecific and defined as pain associated with disability in paediatric age [9]. All the selected articles concluded that rehabilitation interventions were adequate for the outcomes considered.

**Table 6:** Characteristics of included studies

Author, year of publication	Study design	N	Characteristics of the sample	Setting	Duration / intensity of treatment	Follow-up	Effectiveness
*D. K. Brazier* 1997 [17]	Retrospective observational study	/	Age 10–16 Diagnosis: Conversion disorder	Hospital	Duration: 3 weeks Intensity: once a day (time for the session not known)	/	YES
P. Calvert 2003 [16]	Retrospective Case Report	1	Age 14 Diagnosis: SSRD	Hospital Outpatient clinic	Duration: 3 weeks Intensity: two to three times a week (outpatient clinic); four times a week (hospital)	2 months	YES
T.L FitzGerald 2014 [8]	Systematic review	/	Age 0–18 Diagnosis: Conversion disorder	Hospital Outpatient clinic	Hospitalization duration: from 3 days to 16 weeks Therapy duration: from 2.5 weeks to 16 weeks	7 years	YES
M. Gerner 2016 [15]	Retrospective observational study	50	Age 6–18 Diagnosis: DSS	Hospital Outpatient clinic	Outpatient clinic: one to three times a week Hospital: six times a week (time for session not known)	1 year	YES
J.L Gooch 1997 [11]	Retrospective case series	3	Age 9–18 Diagnosis: Conversion disorder	Hospital	Duration: from 3 days to six weeks Intensity not known	/	YES
C.S. Maynard 2010 [9]	Retrospective observational study	41	Age 8–21 Diagnosis: Pain-associated disability syndrome	Hospital	Duration: from four days to seventy-eight days Intensity not known	3 months	YES
G. Mesaroli 2019 [14]	Retrospective Case Report	1	Age 14 Diagnosis: Conversion disorder	Outpatient clinic	Intensity: eight sessions of 1 h each How many times a week not known Duration: three months	/	YES
T.M. Palermo 2001 [10]	Retrospective Case Report	1	Age 11 Diagnosis: DSS	Hospital	Duration: twenty.two days Intensity not known	6 months	YES
G. Ryu 2014 [12]	Retrospective Case Report	1	Age 13 Diagnosis: Conversion disorder	Hospital	Duration: three weeks Intensity: forty minutes, two times a day	2 months	YES

## Results

Table 7 shows the figures who are part of the multidisciplinary team to manage the patient with SSRDs. Except for two studies [14, 17], physiotherapy was described as part of a rehabilitation intervention in which various professional figures participated. Physiotherapists, psychologists, and doctors were always involved in the treatment process [9, 10, 12, 14, 15, 17].

**Table 7:** Members of the multidisciplinary team

Author	Physiotherapist	Occupational Therapist	Psychologist	Doctor	Nurse	Social worker	Teacher	Nutritionist	Not specified
*D. K. Brazier* 1997 [17]	x		x	x	x		x		/
P. Calvert 2003 [16]	/	/	/	/	/	/	/	/	x
M. Gerner 2016 [15]	x	x	x	x	x	x	x		/
J.L Gooch 1997 [11]	/	/	/	/	/	/	/	/	x
C.S. Maynard 2010 [9]	x	x	x	x	x	x	x		/
G. Mesaroli 2019 [14]	x		x	x					/
T.M. Palermo 2001 [10]	x	x	x	x	x	x	x	x	/
G. Ryu 2014 [12]	x		x	x					/

Table 8 shows the outcomes considered by the authors. The restoration of motor functions was the most used [9, 10, 14, 16, 17]; however, only in three studies, it was described with standardized measurement scales such as the Functional Independence Measure (WeeFIM) for children [9], the Functional Disability Inventory (FDI) [10], and the Functional Mobility Scale (FMS) [14]. In the remaining investigations, there was a generic descriptor referring only to the activities proposed in the rehabilitation program [15–17] or within overall assessments of the walk and daily life activities [11, 17]. Only one research [15] evaluated the level of activity and participation as result indicators, according to the International Classification of Functioning, Disability and Health for Children and Youth (ICF-CY). However, the adopted measurement scales were not reported, and the evaluative aspects of the field of body structure analysis were neglected. Two studies measured the quality of life, but only one [10] described the specific questionnaire utilized (Child Health Questionnaire—Parent Form 50). Although the improvement of muscle strength was assumed as a specific short-term goal in three publications, the adopted measurement scale did not appear. [10–12] In another study, improving postural control and balance was proposed as an integral part of the program; nevertheless, the chosen evaluation scales were not included [12].

**Table 8:** Considered outcome

Author	Quality of life	ADL	Muscle strength	Motor skills	Balance	School absenteeism	Sleep quality	Drugs use	Posture	Symptoms’ disappearance	Days of hospitalization	Activity/ partecipation	Standard outcome
M. Gerner [15]										x	?	x	
P. Calvert [16]				x				x		x	?		
T. M. Palermo [10]	x		x	x		x					22 days		FDI; CHQ-PF50
G. Mesaroli [14]				x							/		FMS
L. J. Gooch [11]	x	x	x								9 days – 4 weeks		
G. Ryu [12]			x		x			x	x		?		VAS
D. K. Brazier [17]		x		x							/		
C.S. Maynard [9]				x		x	x	x			4–78 days		WeeFIM

*ADL* Activities of daily living, *FDI* Functional Disability Inventory, *FMS* Functional Mobility Scale, *WeeFIM* Functional Independence Measure for Children, *CHQ-PF50* The Child Health Questionnaire, *VAS* Visual analogue scale

The days of hospitalization, reported by a few authors [9–11], had a wide variability ranging from three to 78 days [9]. In no study, the effectiveness of physiotherapy intervention was evaluated independently from the other rehabilitation proposals by the interdisciplinary team members.

Physiotherapeutic strategies were multiple, but only some studies reported the approach in detail [17](Table 9). Most authors [10, 14–17] proposed gradual exercises of increasing intensity and difficulty, structured so that performance could be rigorously measured. In all the investigations [9–11, 14–18], the importance of establishing clear short-term therapeutic goals (daily/weekly) was emphasized to provide rewarding result feedback to the patients. Empowerment and the therapeutic relationship were considered fundamental ingredients of treatment [8, 10, 14, 16, 17]. In most publications, the general objectives were stated with sufficient clarity. However, only in one study, the operational tools to achieve them were described to be reproduced [17]. In no study was the rationale behind the choices of personalized programs explicit.

**Table 9:** Proposed rehabilitation activities

Author	Graded exercise therapy	Short-term objectives	Gait re-education	Aerobic training	Physical therapy games	Muscle reinforcement	Therapeutic alliance	Stretching	Indipendent exercises	Massages	Re-education activity daily living	Balance exercises	Postural gymnastics
S. C. Maynard [9]		x								x	x		
M. Gerner [15]	x	x											
P. Calvert [16]	x	x	x		x		x						
T. M. Palermo [10]	x	x				x	x						
G. Mesaroli [14]	x	x	x		x		x						
L.J. Gooch [11]		x	x			x						x	x
G. Ryu [12]		x	x			x		x				x	x
D. K. Brazier [17]	x	x	x		x		x		x				

### Evaluation of the methodological quality of research

The study design quality was assessed according to the guidelines indicated on the Equator Network for each type of research considered. Specifically, the following scales were used: STROBE (Strengthening the Reporting of Observational Studies in Epidemiology) for observational studies; PRISMA (Preferred Reporting Items for Systematic Reviews and Meta-Analyses) for revisions; CARE (CAse REports Guidelines) for case reports. The results are shown in Table 10.

**Table 10:** Methodological quality of the studies

Author	Study design	Scale	Title and Abstract	Introduction	Methods	Results	Discussion
M. Gerner [15]	Observational study	STROBE	√	√	/	√	√
P. Calvert et al. [16]	Case Report	CARE	√	√	√	√	√
T. M. Palermo [10]	Case Report	CARE	√	√	√	√	√
G. Mesaroli [14]	Case Report	CARE	/	/	/	/	√
T. L. FitzGerald [8]	Review	PRISMA	√	√	√	√	√
L. J. Gooch [11]	Case Report	CARE	/	√	/	/	√
G. Ryu [12]	Case Report	CARE	/	/	/	/	√
D. K. Brazier [17]	Observational study	STROBE	/	/	/	/	√

All the guidelines checklists used to assess the quality of the studies were divided into the sections such as title and abstract, introduction, methods, results, discussion of the results, and conclusions. The main problems were encountered in the paragraphs dedicated to illustrating the methods and the data processing. Apart from three publications [10, 11, 16] that met all the evaluation criteria, the remaining six were methodologically deficient, especially regarding the description of the study design, the statements of the methodological changes in progress, and the research strategies extraction and processing of the collected data.

No article described the result indicators chosen, nor did it report the evaluation scales used.

The CERT (Consensus on Exercise Reporting Template) guideline, a checklist comprising 16 items, was employed to estimate the quality of physiotherapy treatment reporting. The analysis of the articles demonstrated the poor quality of the description of the treatment programs adopted. No author described the devices (e.g., treadmill, exercise bike), the motivational strategies adopted, the progression of the exercises and their characteristics (repetitions, description of the modalities), or the proposed play activities. The only satisfactory items concerned the setting and description of qualifications, teaching skills, and training carried out by the exercise instructor. Our systematic review considered [8], while stating the lack of clear and precise descriptions of physiotherapy programs in most of the researchers analyzed, did not indicate the evaluation tool used to evaluate the reporting.

## Discussion

This review could not confirm the effectiveness of physiotherapy in the setting of SSD in children and adolescents. Except for two [14, 17], all the reviewed papers considered physiotherapy in the context of the rehabilitation intervention for patients with SSD, which involved various professional figures. Therefore, it was not easy to establish whether and in what terms physiotherapy programs contributed to obtaining the declared results. Furthermore, the study designs, case reports, and observational studies were not appropriate for evaluating the effectiveness of the treatments, rendering the results unreliable. The previous systematic review [8] evaluated only the publications concerning the conversion disorder, concluding that there was no clear evidence of the effectiveness of physiotherapy at the current state of knowledge.

The physiotherapy treatments proposed by the various authors were very heterogeneous and neither reproducible nor comparable due to the lack of reporting quality and declaration of the measurement scales adopted [10, 11, 15, 16]. In addition, in various studies, they were classified as such techniques not specifically within the remit of the physiotherapist. The inclusion of these techniques, such as relaxation strategies [8, 9, 11], cooping [8–10], and the therapeutic relationship [8, 10, 14, 17], resulted in a less rigorous analysis of the results. The importance of attributing the achievement of specific goals to the competent specialist (for example, paediatrician, physiotherapist, occupational therapist, psychologist) was only emphasized in one research [16].

The choice of a rating scale capable of describing all aspects of the clinical manifestations of SSRDs was a critical factor. The ICF-CY was a standardized assessment tool capable of simultaneously contemplating both aspects of body structures and social behaviour. The ICF-CY organized information in two parts: the first dealt with functioning and disability, the second concerning contextual factors, allowing the evaluation of the functioning of the child and adolescent both from an individual and a social perspective. All authors agreed that it was essential to identify long-term goals, choose the correct level of difficulty for the exercises, and establish a solid therapeutic relationship, to achieve adherence to treatment and patient empowerment. No study cited short-term goals; only two detailed the physiotherapy intervention [16, 17], and only one described the device used [17].

In some studies, reference was made to a physiotherapy intervention model called "Restrained rehabilitation" [8, 10, 11, 16] based on a hierarchical scheme of the proposed activities and on the constraint of achieving the objectives shared with the patient for the discharge from the hospital. This approach was inspired by the therapeutic guidelines adopted for chronic fatigue syndrome patients at King's College Hospital [16]. The patient's favourite and most enjoyable activities became a motivating tool, a sort of reward and compensation [8, 16]. The maintenance of results over time was critical in evaluating the effectiveness of the treatment of SSRDs, since it could also relapse months later. Nevertheless, only three authors analyzed a follow-up time of more than three months [9, 10, 15], while others did not consider it [11, 14, 17].

The intensity of the physiotherapy treatment was a critical element impacting its effectiveness. Nevertheless, only one author [16] reported the frequency and duration of the interventions in hospital and outpatient settings. In the remaining articles, it was unclear whether and how the physiotherapy approach adopted during hospitalization was subsequently proposed after discharge [9–12, 17].

By analyzing the length of hospitalization, we noticed a wide variety, ranging from 3 to 78 days. Gooch's study claimed that this difference was based on the heterogeneity of cases detected within a population with the same diagnosis, the conversion disorder, and treated with the same type of rehabilitation intervention. The wide variety and severity of symptomatic manifestations in SSRDs explained, on the one hand, the difficulty in conducting methodologically rigorous randomized controlled trials and, on the other hand, the absence of physiotherapy treatment protocols adaptable to different clinical scenarios.

The most critical aspect in reviewing publications on SSRDs was the rigorous selection of research based on the explicit criteria for the diagnostic definition of SSRDs and the failure to use standardized efficacy measures in research. The SSRDs constituted a new nosological category, inserted in the latest version of the DSM-5, which replaced the previous one of the DSM-IV, in which, for example, the DSS were classified as "somatoform disorders". Therefore, the disorders were categorized according to the previous nosological classifications in many articles, which modified the diagnostic criteria.

Despite the stated limitations, this review highlighted that we need to adopt a more rigorous methodological approach, paying particular attention to the quality of treatment reporting, to evaluate the efficacy of physiotherapy in treating SSRDs. If the proposals were not reproducible due to a lack of data relating to the choice of exercises, their duration, the frequency of the intervention, its possible changes in progress, and if standardized measurement scales were not used, it was practically impossible to compare the results and draw evidence-based conclusions. In the current state of knowledge, it was impossible to establish the effectiveness of the interventions performed and whether and in which clinical contexts one intervention was superior to another. It was essential to define possible physiotherapy protocols whose efficacy could be confirmed later by multicentre randomized controlled studies. SSD is a disorder showing a significant increasing trend, requiring multidisciplinary, time-consuming, costly and demanding treatment in the most severe cases. Although physiotherapy can play an essential role in this context, there is an urgent need to develop rigorous criteria defining the type of interventions and outcomes to establish their actual impact.

## Conclusions

It was not possible to answer the review question regarding the efficacy of physiotherapy interventions in SSRD due to the low number of publications and their lack of methodological quality. Further randomized controlled pilot trials are needed, conducted with a rigorous research methodology, which pays particular attention to the reporting aspect and the evaluation of the effects of physiotherapy treatments independently from those obtained from the other proposals of the multidisciplinary team. Future research should also consider using the ICF-CY as a standardized assessment tool capable of describing outcomes related to the functioning of body structures and the levels of activity and participation of the child and adolescent from both an individual social perspective.

## Data Availability

Not applicable.

## Abbreviations

CARE: CAse REports Guidelines
CERT: Consensus on Exercise Reporting Template
CINAHL: Cumulative Index to Nursing and Allied Health Literature
DSM-5: Diagnostic and Statistical Manual of Mental Disorders 5th edition
FDI: Functional Disability Inventory
FMS: Functional Mobility Scale
ICF-CY: International Classification of Functioning, Disability and Health for Children and Youth
MEDLINE: Medical Literature Analysis and Retrieval System Online
PEDro: Physiotherapy Evidence Database
PRISMA: Preferred Reporting Items for Systematic Reviews and Meta-Analyses
SSD: Somatic Symptom Disorder
SSRDs: Somatic Symptom and Related Disorders
STROBE: STrengthening the Reporting of Observational Studies in Epidemiology
WeeFIM: Functional Independence Measure
